# Medication adherence in hypertension and diabetes comorbidity: implications for disease control in a population-based study

**DOI:** 10.3389/fpubh.2026.1708587

**Published:** 2026-01-30

**Authors:** Yunyun Mei, Hui Zhang, Jingyou Miao, Xinyao Liu, Dingwan Chen, Minmin Jiang, Lilu Ding

**Affiliations:** 1 Department of Epidemiology and Health Statistics, Hangzhou Medical College, Hangzhou, China; 2 Shulan International Medical College, Zhejiang Shuren University, Hangzhou, China

**Keywords:** blood pressure control, diabetes comorbidity, education, hypertension, medication adherence

## Abstract

**Background:**

Medication adherence is essential for effective hypertension management, yet its social and behavioral determinants remain incompletely understood. In particular, the influence of education and comorbid diabetes on adherence is unclear.

**Objectives:**

To examine factors associated with blood pressure control and medication adherence among patients with hypertension, with emphasis on nonlinear educational effects and subgroup differences by sex and comorbidity.

**Methods:**

Using a multistage random sampling design, we recruited 40,037 adults with physician-diagnosed hypertension across all 11 prefecture-level cities in Zhejiang. Participants were identified through electronic chronic disease registries and completed standardized questionnaires. Blood pressure control was defined according to national guidelines, and medication adherence was measured by self-reported consistent use of prescribed antihypertensive medication in the past 2 weeks. Multivariable logistic regression models were fitted to identify associated factors, and restricted cubic spline and quadratic models were applied to assess nonlinear education–adherence relationships.

**Results:**

Medication adherence strongly predicted hypertension control (aOR = 1.35, 95% CI: 1.25–1.46). Adherence was higher among patients with comorbid diabetes (aOR = 1.52, 95% CI: 1.39–1.66) and those with higher income, but declined at the highest education levels. Nonlinear analyses revealed inverted U-shaped associations, with thresholds around 4–8 years of schooling. These patterns differed by sex and comorbidity status.

**Conclusion:**

Education exerts threshold-dependent effects on adherence, modified by sex and diabetes comorbidity. By uncovering nonlinear and subgroup-specific patterns, this study extends prior evidence and underscores the need for tailored interventions to reduce disparities in hypertension management.

## 1 Introduction

Chronic non-communicable diseases (NCDs) have become a defining public health challenge of the 21st century, driven by rapid population ageing and widespread lifestyle transitions (1). Among them, hypertension and type 2 diabetes are the most prevalent and burdensome conditions worldwide (2). Their coexistence substantially increases the risk of cardiovascular and cerebrovascular diseases, renal dysfunction, and premature mortality (3, 4). The Global Burden of Disease Study 2019 estimated that 1.28 billion people lived with hypertension, with over three-quarters residing in low- and middle-income countries, while the global prevalence of diabetes reached 460 million (5). The rising comorbidity of these two conditions has further amplified the complexity and cost of chronic disease management.

The dual burden of hypertension and diabetes not only poses challenges for clinical control but also intensifies healthcare resource demands and economic strain on patients and families. Pharmacological therapy remains the cornerstone of management, yet its effectiveness relies heavily on patient adherence. Evidence indicates that optimal adherence can reduce cardiovascular risk by approximately 20% (6, 7). However, adherence remains suboptimal: global rates rarely exceed 50%, and in China, only 24% of hypertensive patients achieve adequate blood pressure control, while glycaemic control among patients with diabetes is similarly poor (8, 9).

Given its pivotal role in disease control, adherence has been the focus of increasing research attention. Prior studies have linked adherence to sociodemographic characteristics, socioeconomic status, health literacy, lifestyle behaviours, and the physician–patient relationship (10–13). Despite these advances, several critical gaps remain. First, most analyses have assumed linear associations between predictors and adherence, offering little insight into potential non-linear effects or thresholds in social determinants. Second, systematic investigations of adherence among patients with both hypertension and diabetes are scarce, leaving important questions about multimorbidity management unanswered. Third, patterns of adherence and their determinants across population subgroups, such as by sex or age, remain underexplored.

To address these gaps, we conducted a large-scale, population-based cross-sectional survey in Zhejiang Province, China. We assessed medication adherence among patients with hypertension and diabetes, examined its relationship with disease control, and explored the multidimensional influences of socioeconomic, demographic, and health behavioural factors. Importantly, we applied flexible modelling approaches, including restricted cubic splines and quadratic regression models, to capture potential non-linear effects and subgroup heterogeneity. By doing so, this study aims to generate robust empirical evidence to inform more targeted and stratified intervention strategies for chronic disease management.

## 2 Materials and methods

### 2.1 Study design and population

This population-based cross-sectional study was conducted in Zhejiang Province, China, and reported in accordance with the Strengthening the Reporting of Observational Studies in Epidemiology (STROBE) guidelines (14). A multistage, stratified random sampling strategy was used to ensure representativeness of patients enrolled in chronic disease management programs across both urban and rural areas.

Eligible participants were permanent residents of Zhejiang Province aged ≥18 years with a confirmed diagnosis of hypertension recorded in a qualified medical institution and currently receiving antihypertensive medication. Individuals were excluded if they had severe cognitive impairment or psychiatric disorders preventing informed consent or reliable participation, secondary or pregnancy-related hypertension, or missing/inconsistent responses on key variables. Written informed consent was obtained from all participants.

The required sample size was calculated to achieve precise prevalence estimates and sufficient power for multivariable analyses. Assuming a conservative adherence prevalence of 50%, a 95% confidence level, and a 0.5% margin of error (15), the minimum required sample was 38,416. After adjusting for a design effect of 1.05 and an anticipated 5% nonresponse rate, the operational target was ~42,400 participants.

To operationalize this target, we implemented a multistage random sampling strategy aligned with the administrative hierarchy of Zhejiang Province. In the first stage, all 11 prefecture-level cities were included to ensure complete geographic coverage. In the second stage, one urban district and one rural county were randomly selected within each city, yielding 22 primary sampling units (PSUs). In the third stage, the target sample was proportionally allocated across PSUs according to their population size. Within each PSU, participants were randomly selected from local electronic chronic disease management registries.

If a PSU lacked sufficient eligible individuals to fulfill its assigned quota, the shortfall was reallocated to adjacent units within the same city, and additional random draws were conducted. To ensure quality and reduce selection bias, randomization was centrally supervised, registry lists were cross-validated against local health authority records, and data collection was monitored by provincial coordinators with routine checks for completeness and consistency. The final analytic sample comprised 40,037 valid responses, exceeding the minimum required size and ensuring robust statistical power for regression and subgroup analyses.

### 2.2 Questionnaire design

The questionnaire was developed to capture the management needs of patients with hypertension and diabetes, drawing on the national chronic disease surveillance framework, published literature, and expert consultation (16–18). Following pilot testing, the final instrument included four domains: demographic information, household and socioeconomic status, disease history, and utilization of chronic disease management services. The primary outcome was blood pressure control, defined as systolic blood pressure <140 mmHg and diastolic blood pressure <90 mmHg at the time of survey measurement, in accordance with the Seventh Report of the Joint National Committee on Prevention, Detection, Evaluation, and Treatment of High Blood Pressure (19).

Blood pressure measurements were conducted on-site by trained primary healthcare staff using validated automated sphygmomanometers. Participants were seated and rested for at least 5 min prior to measurement. Blood pressure was measured on the upper arm using an appropriately sized cuff. Two consecutive measurements were obtained during the survey visit, and the average of the two readings was used to determine blood pressure control status.

A secondary outcome was medication adherence, defined as self-reported consistent use of prescribed antihypertensive medication in the past 2 weeks. Specifically, antihypertensive medication adherence was measured using the following item: “If you take antihypertensive medication, how is your adherence? “Response options included: (1) Regular, (2) Intermittent, and (3) Do not take medication. Participants who selected “Regular” were classified as adherent, whereas those reporting “Intermittent” or “Do not take medication” were classified as non-adherent. This item corresponds to Question B1.3 in the questionnaire and was administered to all participants with physician-diagnosed hypertension. The two-week recall period was chosen to balance recall accuracy and clinical relevance. The full questionnaire, including all items, response options, and skip patterns, is provided as Supplementary Table S1. Diabetes status was determined based on self-reported physician diagnosis; participants reporting a prior diagnosis of diabetes by a healthcare professional were classified as having diabetes.

Covariates included sociodemographic and clinical characteristics: gender (female as reference), age (continuous, in years), ethnicity (Han as reference), education level (no formal schooling as reference; primary and below, junior high, high school, college and above), occupation (retired as reference; jobless/unemployed, household labor, commerce/business, farmers, other practitioners), marital status (unmarried as reference; married, divorced, widowhood), living arrangement (living alone as reference; not living alone), health insurance (basic medical insurance for urban workers as reference; medical insurance for urban and rural residents, commercial medical insurance, no health insurance), and annual household income (<30,000 RMB as reference; 30,000–79,999 RMB, ≥80,000 RMB). Patient type was also considered (hypertension only as reference; hypertension with diabetes).

### 2.3 Data sources

Data were collected through on-site surveys conducted from March 2021 to October 2022 at primary healthcare facilities across selected sampling points. Trained investigators administered the standardized questionnaire. Data quality was ensured through double-entry verification, logical consistency checks, and oversight by provincial coordinators. The analytic dataset was anonymized and stored in an encrypted database in compliance with the Declaration of Helsinki and China’s Personal Information Protection regulations (20–22).

### 2.4 Statistical analysis

Categorical variables were summarized as frequencies and percentages and compared using the chi-square test. Continuous variables were expressed as means ± standard deviations (SD). Normality was assessed prior to group comparisons, and the Mann–Whitney *U*-test was applied when normality assumptions were violated.

Hypertension control and medication adherence were modeled as dependent variables in multivariable logistic regression analyses (34). Although a multistage stratified sampling design was used for participant recruitment, regression analyses focused on examining associations between variables rather than producing population-level prevalence estimates. Therefore, sampling weights were not applied in the regression models. Given the large sample size and proportional allocation across primary sampling units, the impact of ignoring sampling weights on effect estimates is expected to be limited.

For adherence, sequential models (Models 1–3) were constructed to test robustness after adjustment for potential confounders. Model 1 adjusted for demographic and socioeconomic characteristics; Model 2 further adjusted for lifestyle-related behaviors; and Model 3 additionally adjusted for family history of chronic diseases. Adjusted odds ratios (aORs) with 95% confidence intervals (CIs) were reported. Potential nonlinear associations between years of education and medication adherence were examined using restricted cubic spline analyses to assess overall trends. Quadratic logistic regression models including both linear and squared terms for education were further fitted in subgroup analyses for visualization and estimation of turning points. Turning points were analytically derived from the quadratic regression coefficients. To quantify uncertainty, bootstrap resampling with 1,000 iterations (seed = 123) was performed, and 95% confidence intervals were constructed using the percentile method. Missing values in annual household income were handled using multiple imputation by chained equations (MICE) with predictive mean matching (PMM). Five imputations were generated (m = 5; seed = 1,024) using the mice package in R. Imputed values were used to construct the analytical dataset for subsequent regression analyses. All analyses were conducted in R version 4.3.1 (R Foundation for Statistical Computing, Vienna, Austria) (23).

## 3 Results

A total of 40,037 patients with hypertension were included in the final analysis. Of these, 23,851 (59.6%) had hypertension only and 16,186 (40.4%) had comorbid diabetes. The mean age was 68.9 ± 9.46 years, and 42.4% were male. Baseline sociodemographic and socioeconomic characteristics differed significantly between the two groups (Table 1). Compared with patients with isolated hypertension, those with comorbid diabetes had lower educational attainment (junior high school or above: 27.5% vs. 30.0%, *p* < 0.001), a slightly higher retirement rate (21.4% vs. 20.5%, *p* < 0.001), and a higher proportion with annual income ≥80,000 RMB (12.9% vs. 11.7%, *p* < 0.001).

**Table 1:** Baseline characteristics of patients with hypertension, with and without comorbid diabetes.

Characteristics	Patients with hypertension (*N* = 23,851)	Patients with hypertension and diabetes (*N* = 16,186)	*p*
Gender			**<0.001**
Male	10,423 (43.7%)	6,536 (40.4%)	
Female	13,428 (56.3%)	9,650 (59.6%)	
Age			0.092
Mean (SD)	68.9 (9.46)	69.0 (8.66)	
Missing	31 (0.1%)	25 (0.2%)	
Ethnicity			0.714
Han	23,724 (99.5%)	16,105 (99.5%)	
Other	127 (0.5%)	81 (0.5%)	
Education level			**<0.001**
No formal schooling	5,629 (23.6%)	4,165 (25.7%)	
Primary and below	11,053 (46.3%)	7,575 (46.8%)	
Junior high school	5,092 (21.3%)	3,183 (19.7%)	
High school	1,502 (6.3%)	932 (5.8%)	
College and above	575 (2.4%)	331 (2.0%)	
Occupation			**<0.001**
Retired	4,887 (20.5%)	3,466 (21.4%)	
Jobless or unemployed	2,586 (10.8%)	1866 (11.5%)	
Household labor	6,753 (28.3%)	5,167 (31.9%)	
Commercial/business	614 (2.6%)	381 (2.4%)	
Farmer	7,180 (30.1%)	4,335 (26.8%)	
Other practitioners	1831 (7.7%)	971 (6.0%)	
Marital status			**<0.001**
Unmarried	263 (1.1%)	172 (1.1%)	
Married	20,178 (84.6%)	13,474 (83.2%)	
Divorce	207 (0.9%)	180 (1.1%)	
Widowhood	3,203 (13.4%)	2,360 (14.6%)	
Living arrangement			**<0.001**
Living alone	3,501 (14.7%)	2,580 (15.9%)	
Not living alone	20,350 (85.3%)	13,606 (84.1%)	
Health insurance			**<0.001**
Urban workers’ insurance	6,118 (25.7%)	4,444 (27.5%)	
Urban–rural residents’ insurance	17,555 (73.6%)	11,645 (71.9%)	
Commercial medical insurance	49 (0.2%)	42 (0.3%)	
No health insurance	129 (0.5%)	55 (0.3%)	
Annual household income			**<0.001**
<30,000 RMB	12,379 (51.9%)	8,561 (52.9%)	
30,000–79,999 RMB	8,670 (36.4%)	5,540 (34.2%)	
≥80,000 RMB	2,802 (11.7%)	2,085 (12.9%)	

Bold *P* values indicate statistical significance (*P* < 0.05).

In multivariable logistic regression analyses, blood pressure control was associated with demographic, socioeconomic, and behavioral characteristics (Table 2). Higher education (college or above: aOR = 2.06, 95% CI: 1.75–2.44) and higher income (≥80,000 RMB: aOR = 1.20, 95% CI: 1.12–1.28) were positively associated with control. Compared with retirees, lower control rates were observed among participants engaged in agricultural work (aOR = 0.65, 95% CI: 0.60–0.69), commerce/business (aOR = 0.75, 95% CI: 0.65–0.86), and household work (aOR = 0.69, 95% CI: 0.65–0.75) (all *p* < 0.001). Medication adherence was also positively associated with blood pressure control (high adherence: aOR = 1.35, 95% CI: 1.25–1.46).

**Table 2:** Factors associated with blood pressure control in multivariable logistic regression.

Characteristics	aOR	95% CI	*p*
Gender			
Female	—	—	
Male	0.95	0.91, 1.00	0.046
Age	1.01	0.99, 1.01	**<0.001**
Ethnicity			
Han	—	—	
Other	1.10	0.83, 1.45	0.5
Education level			
No formal schooling	—	—	
Primary and below	1.23	1.16, 1.29	**<0.001**
Junior high school	1.26	1.18, 1.35	**<0.001**
High school	1.53	1.39, 1.70	**<0.001**
College and above	2.06	1.75, 2.44	**<0.001**
Occupation			
Retired	—	—	
Jobless/unemployed	0.73	0.67, 0.79	**<0.001**
Household labor	0.69	0.65, 0.75	**<0.001**
Commercial/business	0.75	0.65, 0.86	**<0.001**
Farmer	0.65	0.60, 0.69	**<0.001**
Other practitioners	0.95	0.86, 1.04	0.2
Marital status			
Unmarried	—	—	
Married	0.90	0.74, 1.10	0.3
Divorce	0.95	0.72, 1.27	0.7
Widowhood	0.83	0.68, 1.02	0.077
Living arrangement			
Living alone	—	—	
Not living alone	1.09	1.02, 1.16	**0.011**
Health insurance			
Urban workers’ insurance	—	—	
Urban–rural residents’ insurance	0.82	0.77, 0.87	**<0.001**
Commercial medical insurance	0.69	0.45, 1.05	0.083
No health insurance	1.11	0.82, 1.51	0.5
Annual household income			
<30,000 RMB	—	—	
30,000–79,999 RMB	1.19	1.13, 1.24	**<0.001**
≥80,000 RMB	1.20	1.12, 1.28	**<0.001**
Patient type			
Hypertension only	—	—	
Hypertension with diabetes	1.01	0.97, 1.06	0.5
Medication adherence	1.35	1.25,1.46	**<0.001**

Bold *P* values indicate statistical significance (*P* < 0.05).

**Figure 1:**
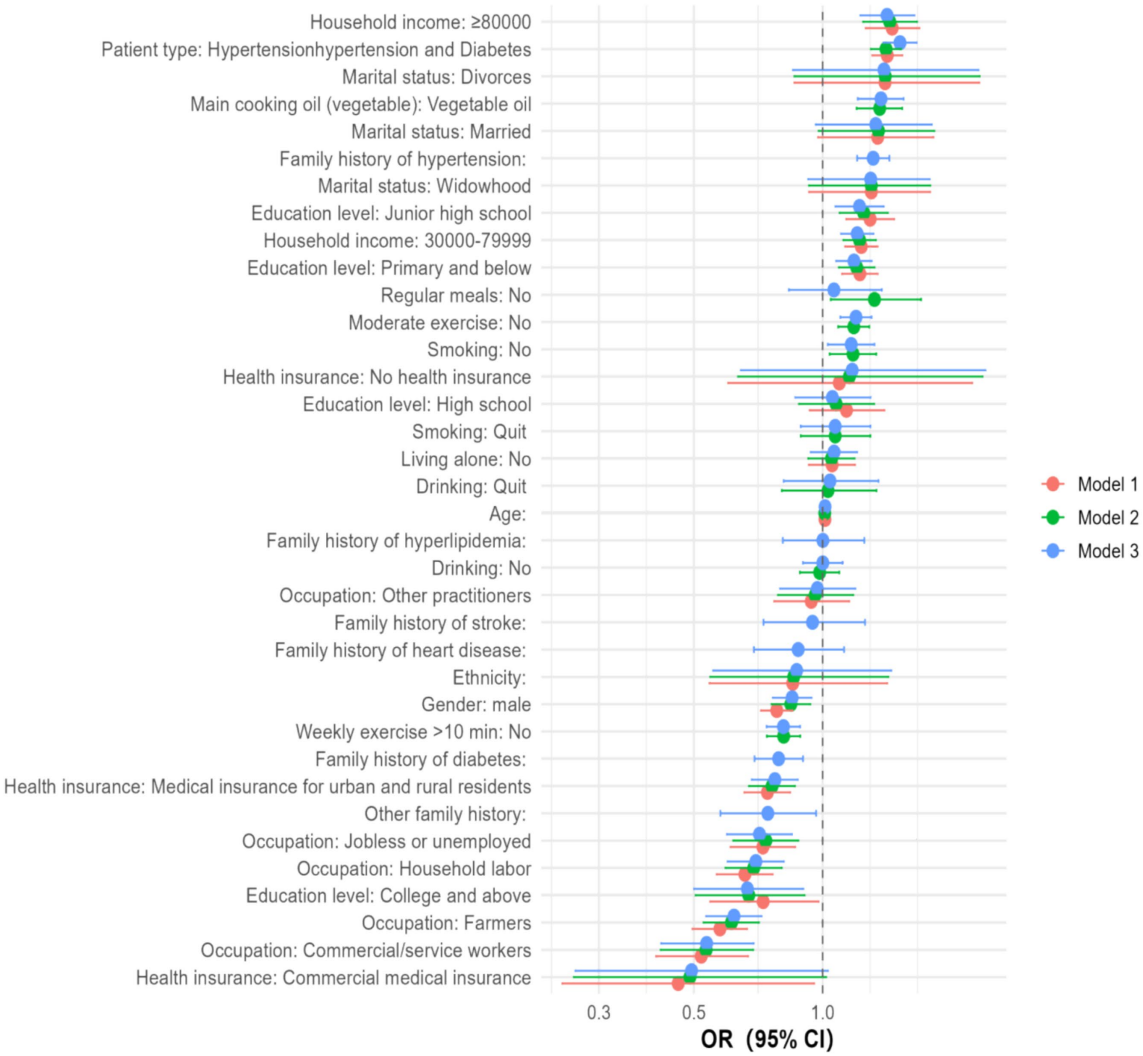
Factors associated with medication adherence: multivariable logistic regression (odds ratios and 95% CIs). Nonlinear analyses revealed a threshold effect between years of education and adherence (Figures 2–4). Restricted cubic spline and quadratic models indicated an inverted U–shaped relationship, with subgroup-specific turning points. Among men, adherence peaked at 7.71 years of schooling (95% CI: 6.37–9.51), while among women the peak occurred earlier, at 6.17 years (95% CI: 5.19–7.78). In comorbidity subgroups, the turning point was estimated at 7.70 years (95% CI: 5.78–13.93) for patients with isolated hypertension and at 4.23 years (95% CI: 0.48–7.05) for those with comorbid diabetes.

Key correlates of adherence were identified (Figure 1; Table 3). Participants with comorbid diabetes had higher adherence than those with isolated hypertension (aOR = 1.52, 95% CI: 1.39–1.66). Higher income was associated with better adherence (aOR = 1.41, 95% CI: 1.23–1.64). By contrast, individuals with college education or above were less likely to adhere (aOR = 0.67, 95% CI: 0.50–0.90). Health behaviors, such as regular physical activity and scheduled meals, showed only modest associations with adherence, but these positive effects disappeared after full adjustment, suggesting that their potential role warrants further investigation.

**Table 3:** Factors associated with medication adherence in multivariable logistic regression.

Characteristic	Model 1			Model 2			Model 3		
	aOR	95% CI	*p*	aOR	95% CI	*p*	aOR	95% CI	*p*
Gender									
Female	—	—		—	—		—	—	
Male	0.78	0.72, 0.85	**<0.001**	0.84	0.76, 0.94	**0.001**	0.85	0.76, 0.94	**0.002**
Age	1.01	1.01, 1.02	**<0.001**	1.01	1.01, 1.02	**<0.001**	1.01	1.01, 1.02	**<0.001**
Ethnicity									
Han	—	—		—	—		—	—	
Other	0.85	0.54, 1.42	0.508	0.86	0.55, 1.42	0.523	0.87	0.55, 1.45	0.567
Education level									
No formal schooling	—	—		—	—		—	—	
Primary and below	1.22	1.11, 1.34	**<0.001**	1.20	1.09, 1.32	**<0.001**	1.18	1.07, 1.30	**<0.001**
Junior high school	1.29	1.14, 1.47	**<0.001**	1.25	1.10, 1.42	**<0.001**	1.22	1.07, 1.39	**0.003**
High school	1.14	0.93, 1.39	0.210	1.08	0.88, 1.32	0.483	1.05	0.86, 1.29	0.611
College and above	0.73	0.55, 0.98	**0.031**	0.67	0.50, 0.91	**0.008**	0.67	0.50, 0.90	**0.007**
Occupation									
Retired	—	—		—	—		—	—	
Jobless or unemployed	0.73	0.61, 0.86	**<0.001**	0.74	0.62, 0.88	**<0.001**	0.71	0.60, 0.85	**<0.001**
Household labor	0.66	0.57, 0.76	**<0.001**	0.69	0.59, 0.80	**<0.001**	0.70	0.60, 0.81	**<0.001**
Commercial/business	0.52	0.41, 0.67	**<0.001**	0.53	0.42, 0.69	**<0.001**	0.54	0.42, 0.69	**<0.001**
Farmer	0.58	0.50, 0.67	**<0.001**	0.61	0.53, 0.71	**<0.001**	0.62	0.53, 0.72	**<0.001**
Other practitioners	0.94	0.77, 1.15	0.554	0.96	0.79, 1.18	0.702	0.97	0.80, 1.19	0.785
Marital status									
Unmarried	—	—		—	—		—	—	
Married	1.35	0.98, 1.82	0.061	1.35	0.98, 1.83	0.058	1.33	0.96, 1.80	0.072
Divorce	1.40	0.86, 2.33	0.184	1.40	0.86, 2.33	0.183	1.39	0.85, 2.31	0.193
Widowhood	1.30	0.93, 1.78	0.114	1.30	0.93, 1.78	0.114	1.30	0.93, 1.78	0.120
Living arrangement									
Living alone	—	—		—	—		—	—	
Not living alone	1.05	0.93, 1.19	0.421	1.05	0.93, 1.19	0.449	1.06	0.94, 1.20	0.335
Health insurance									
Urban workers’ insurance	—	—		—	—		—	—	
Urban–rural residents’ insurance	0.74	0.66, 0.84	**<0.001**	0.76	0.67, 0.86	**<0.001**	0.77	0.68, 0.87	**<0.001**
Commercial medical insurance	0.46	0.25, 0.96	**0.023**	0.49	0.25, 0.96	**0.037**	0.49	0.26, 0.96	**0.040**
No health insurance	1.09	0.60, 2.24	0.786	1.16	0.63, 2.36	0.664	1.17	0.64, 2.40	0.632
Annual household income									
<30,000 RMB	—	—		—	—		—	—	
30,000–79,999 RMB	1.23	1.13, 1.34	**<0.001**	1.22	1.12, 1.33	**<0.001**	1.20	1.10, 1.32	**<0.001**
≥80,000 RMB	1.45	1.26, 1.68	**<0.001**	1.44	1.24, 1.66	**<0.001**	1.41	1.23, 1.64	**<0.001**
Patient type									
Hypertension only	—	—		—	—		—	—	
Hypertension with diabetes	1.42	1.31, 1.54	**<0.001**	1.41	1.30, 1.53	**<0.001**	1.52	1.39, 1.66	**<0.001**
Smoking									
Yes				—	—		—	—	
No				1.18	1.04, 1.34	**0.011**	1.17	1.03, 1.32	**0.017**
Quit smoking				1.07	0.89, 1.29	0.477	1.07	0.89, 1.29	0.479
Drinking									
Yes				—	—		—	—	
No				0.98	0.88, 1.09	0.771	1.00	0.90, 1.11	0.982
Quit drinking				1.03	0.80, 1.34	0.822	1.04	0.81, 1.35	0.761
Exercise moderate strength									
Yes				—	—		—	—	
No				1.18	1.09, 1.29	**<0.001**	1.20	1.10, 1.30	**<0.001**
Exercise sports longer than 10 min									
Yes				—	—		—	—	
No				0.81	0.74, 0.89	**<0.001**	0.81	0.74, 0.89	**<0.001**
Regular three meals									
Yes				—	—		—	—	
No				1.32	1.04, 1.70	**0.024**	1.06	0.83, 1.38	0.633
Main cooking oil									
Animal oil				—	—		—	—	
Vegetable oil				1.36	1.20, 1.54	**<0.001**	1.37	1.21, 1.55	**<0.001**
Family history stroke							0.95	0.73, 1.26	0.696
Family history diabetes							0.79	0.69, 0.90	**<0.001**
Family history hypertension							1.31	1.20, 1.43	**<0.001**
Family history hyperlipidemia							1.00	0.81, 1.25	0.990
Family history heart attack							0.88	0.69, 1.12	0.288
Family history other							0.74	0.58, 0.97	**0.025**

Model 1 adjusted for demographic and socioeconomic characteristics; Model 2 additionally adjusted for lifestyle-related behaviors; Model 3 further adjusted for family history of chronic diseases; Bold *P* values indicate statistical significance (*P* < 0.05).

**Figure 2:**
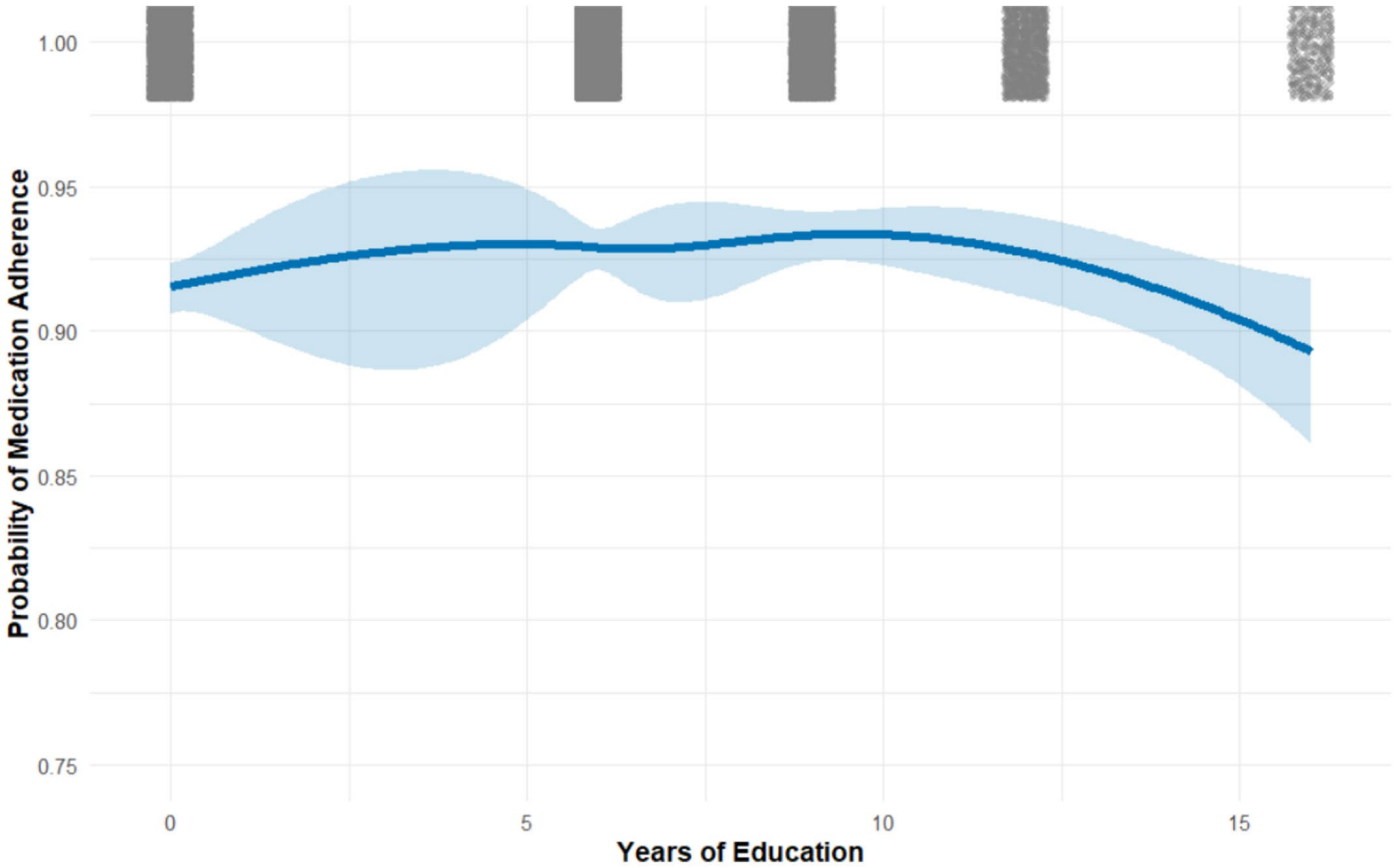
Nonlinear association between years of education and medication adherence among patients with hypertension (restricted cubic spline model).

**Figure 3:**
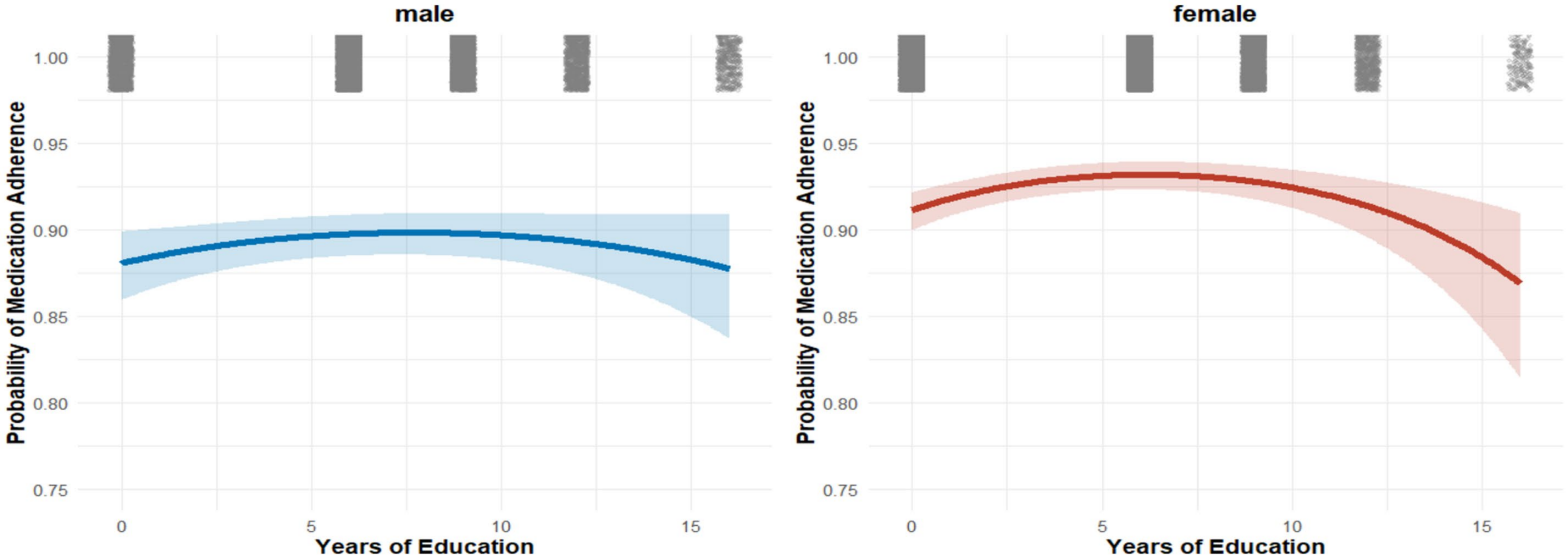
Sex-specific nonlinear associations between years of education and medication adherence (quadratic model).

**Figure 4:**
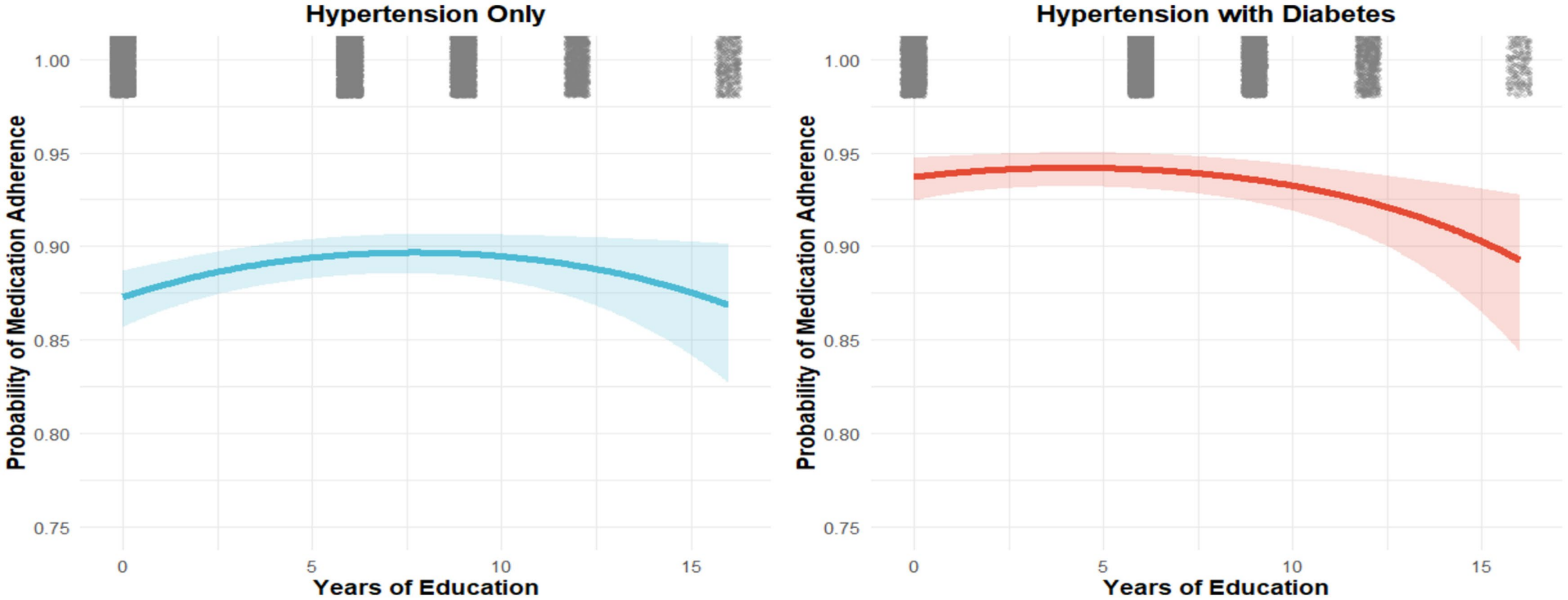
Nonlinear associations between years of education and medication adherence stratified by comorbid diabetes status (quadratic model).

## 4 Discussion

In this large, population-based study of over 40,000 patients with hypertension in Zhejiang Province, we observed distinct patterns in blood pressure control and medication adherence, shaped by socioeconomic, occupational, and behavioral factors. Several important insights emerge. Higher educational attainment and household income were consistently associated with better blood pressure control, underscoring the role of socioeconomic resources in chronic disease management. Medication adherence also showed a robust independent association with blood pressure control, underscoring its central role in hypertension management. Importantly, we identified a nonlinear, threshold-dependent relationship between education and adherence, with adherence improving at lower education levels but diminishing beyond specific turning points, particularly among women and patients with comorbid diabetes. Together, these findings suggest that adherence-promoting strategies should be context-sensitive and tailored to patients’ social and clinical profiles.

Our observation that education and income facilitate better blood pressure control aligns with prior studies demonstrating that socioeconomic advantage enhances health literacy, access to care, and treatment continuity (24, 25). For example, national surveys in China and elsewhere consistently report higher rates of hypertension control among individuals with greater education and income levels (26). However, our adherence analyses suggest that this relationship is not straightforward. At lower education levels, each additional year of schooling was associated with higher adherence, likely reflecting improved health awareness and receptivity to medical guidance. Yet beyond turning points—approximately 6–8 years for most groups—further education was paradoxically linked to lower adherence. This inverted U-shaped pattern challenges the conventional assumption that “more education always translates to better adherence.”

Several mechanisms may underlie this counterintuitive effect. More highly educated patients may exercise greater autonomy, critically appraise medical advice, and adopt alternative health practices, thereby reducing strict adherence to prescribed regimens (27). Among patients with comorbid diabetes, the effect was particularly pronounced, with earlier and sharper declines in adherence at higher education levels. This may reflect the added burden of polypharmacy, health-related anxiety, or decision fatigue from managing multiple conditions simultaneously (28). By contrast, less educated patients may be more inclined to follow physicians’ instructions closely, especially within China’s primary-care–based chronic disease management system (29). These nuanced findings underscore the importance of considering not only socioeconomic gradients but also nonlinear and heterogeneous effects when designing adherence interventions.

The sex-specific differences we identified further enrich this picture. Women showed steeper adherence gains at lower education levels, but adherence declined more sharply after the peak compared with men. This suggests that educational attainment may interact with gender roles, health perceptions, and social expectations, creating distinct adherence trajectories (30). Similar gender heterogeneity has been reported in other chronic disease contexts, though rarely characterized in a nonlinear framework (31–33). Our findings thus extend the literature by providing empirical evidence of threshold-dependent effects that vary by sex and comorbidity.

These results have important practical implications. Interventions aiming to improve adherence promotion strategies should move beyond a one-size-fits-all models and explicitly address heterogeneity by sex, education, and comorbidity status. For patients with lower education, interventions might focus on strengthening health literacy and building trust in medical guidance. For patients with lower education, interventions should focus on strengthening health literacy and building trust in medical guidance, whereas for those with higher education—particularly women and individuals with diabetes—tailored counseling and shared decision-making may help mitigate decision fatigue. Encouraging patient involvement in personalized care monitoring and follow-up would further enhance adherence and engagement across these subgroups. Because these determinants interact in complex ways, Future interventions should adopt a multidisciplinary approach that incorporates nutritional assessment, physical activity promotion, and psychological support, tailored to age and comorbidity profiles. Such integrative strategies could help sustain long-term blood pressure control beyond pharmacological management.

In addition, the strong association between socioeconomic resources and both adherence and blood pressure control highlights the need to reduce financial and structural barriers to consistent medication use. The observed links between healthier lifestyle behaviors and better adherence also suggest that integrated interventions promoting physical activity and dietary regularity could yield synergistic benefits.

### 4.1 Strengths of study

This study has several strengths. First, it leveraged a large sample of patients with hypertension from Zhejiang Province, providing substantial statistical power to detect subgroup heterogeneity and to evaluate nonlinear associations. The population-based design and multistage sampling framework increase the credibility of the observed patterns within the study setting and reduce the risk that results are driven by small-sample instability.

Second, we applied advanced statistical approaches to interrogate nonlinear relationships and effect modification, enabling a more realistic characterization of how education relates to adherence across the attainment spectrum and across clinically relevant subgroups. By moving beyond linear assumptions, our analyses provide actionable insights for targeted intervention design and help reconcile seemingly inconsistent findings in the prior literature that may have been driven by model misspecification.

### 4.2 Limitations

Despite these strengths, several limitations must be considered. From a perspective of study design, the cross-sectional nature of our data precludes the establishment of temporal precedence, raising the possibility of reverse causation. For instance, while we suggest that education influences adherence, it is also possible that patients with poor health outcomes (due to low adherence) interact more frequently with the healthcare system, which in turn alters their health-seeking behaviors and reported adherence levels. Furthermore, although we adjusted for various socioeconomic and behavioral factors, the potential for residual confounding remains. Unmeasured variables, such as the specific quality of patient-physician communication, psychological resilience, or the out-of-pocket cost of medications at different local clinics, could further explain the variance in BP control.

Selection bias and data representation also present challenges. While our sampling was rigorous, the exclusion of individuals with incomplete records or those unable to attend the survey visit may lead to an underrepresentation of the most frail or highly mobile populations, potentially biasing our estimates of control rates. Regarding our statistical estimates, although we utilized a large sample, the absence of complex sampling weights in certain sub-analyses means that the results primarily reflect the studied cohort rather than a perfectly weighted projection of the entire provincial population. Finally, the reliance on self-reported adherence introduces inherent recall and social desirability biases. Patients may overstate their adherence to align with perceived medical expectations. While our use of standardized BP measurements mitigates some of this subjectivity, future research should aim to incorporate objective metrics, such as pharmacy refill records or digital pill-bottle monitoring, to validate these self-reported patterns. Additionally, blood pressure control was defined based on the average of two measurements obtained during the same survey visit, which may not accurately reflect long-term control patterns that require repeated assessments.

## 5 Conclusion

This study demonstrates that socioeconomic, occupational, and behavioral factors play critical roles in hypertension management, with medication adherence serving as a central determinant of blood pressure control. Importantly, we uncover nonlinear, threshold-dependent effects of education on adherence, moderated by sex and comorbidity status. These findings highlight the need for precision in adherence promotion—tailoring interventions not only to socioeconomic gradients but also to the specific turning points where additional education ceases to confer benefit and may even undermine adherence. Such nuanced approaches are essential for improving chronic disease outcomes in increasingly diverse and complex patient populations.

## Data Availability

The raw data supporting the conclusions of this article will be made available by the authors, without undue reservation.
