# Supplementary material for: Medication adherence in hypertension and diabetes comorbidity: implications for disease control in a population-based study
Source: Front Public Health. 2026 Jan 30;14:1708587. doi: 10.3389/fpubh.2026.1708587 (PMC12901457; doi:10.3389/fpubh.2026.1708587)
Supplement: Supplementary file 1 [file Table_1.DOCX]

**Survey on the Life and Health of Patients with Two Chronic Diseases**

**□ Hypertension only □Diabetes only**

**□Hypertension combined with Diabetes**

Hello! Welcome to our survey! In order to support the reform work on the “two chronic diseases,” we are conducting this baseline survey. Please answer truthfully according to your own knowledge. Thank you!

| **ID nember：□□□□□□□□□□□□□□□□** |
| --- |
| **Name： Phone number：_________________** |
| **Residential Address: Township(Street)**  **Village(Neighborhood)** |
| **Investigator’s signature**：___________ **Date completed**：_______  **Reviewer’s signature**：___________ **Date completed**：_______ |

| **Part I. Basic Information** | | |
| --- | --- | --- |
| A1 | **Ethnicity:** (1) Han (2) Other |  |
| A2 | **Education level:** (1) No formal schooling (2) Primary school or below (3) Junior high school (4) High school/technical secondary/vocational school (5) Junior college  (6) Bachelor’s degree or above |  |
| A3 | **Occupation:** (1) Retired (2) Unemployed (3) Housework (4) Government/enterprise manager (5) Professional/technical staff (6) General staff (7) Business/service worker   1. Military personnel (9) Non-agricultural industrial worker (10) Farmer engaged in non-agricultural labor 2. (11) Agricultural worker (farming, forestry, animal husbandry, fishery) (12) Other |  |
| A4 | **Marital status:** (1) Single (2) Married (3) Divorced (4) Widowed |  |
| A5 | **Do you currently live alone?** (1) Yes (2) No |  |
| A6 | **What type of medical insurance do you mainly have?**  (1) Urban employee basic medical insurance (2) Urban–rural resident medical insurance (3) Commercial medical insurance (4) None |  |
| A7 | **What was your household’s average annual income in the past year (including children and elderly living together)?** (1) < 10,000 yuan (2) 10,000–29,999 yuan (3) 30,000–49,999 yuan (4) 50,000–79,999 yuan (5) 80,000–149,999 yuan (6) ≥150,000 yuan (9) Prefer not to answer |  |
| **Part II. Major Chronic Disease History and Treatment** | | |
| B | **Do you have a family history of the following diseases?** (Multiple choice except option 7) (1) Stroke (2) Diabetes (3) Hypertension (4) Hyperlipidemia (5) Heart disease (6) Other (please specify) (7) None |  |
| **Hypertension** | | |
| B1 | **Have you ever been diagnosed with hypertension by a doctor at a formal medical institution?** (1) Yes (2) No (skip to B2) (9) Don’t know (skip to B2) |  |
| B1.1 | **Year of hypertension diagnosis:** ____ |  |
| B1.2 | **Which of the following measures do you take to control blood pressure?** (Multiple choice except option 1) (1) None (2) Take antihypertensive medication (3) Dietary control (4) Reduce salt intake  (5) Increase exercise (6) Blood pressure monitoring (7) Other (please specify) |  |
| B1.3 | **If you take antihypertensive medication, how is your adherence?** (1) Regular (skip to B1.5) (2) Intermittent (3) Do not take medicine |  |
| B1.4 | **If not regular, what is the main reason?** (1) Didn’t know medication is necessary (2) Unwilling, think it doesn’t matter (3) Too expensive (4) Side effects or adverse reactions (5) Inconvenient to buy medication (6) Stop when BP normalizes (7) Other |  |
| B1.5 | **Have you ever received guidance from a doctor or nurse on antihypertensive medication?** (1) Yes (2) No |  |
| B1.6 | **Do you monitor your blood pressure at home?** (1) Rarely (2) Occasionally (<1/day) (3) Regularly 1–3 times/day (4) More than 3 times/day |  |
| B1.7 | **After diagnosis, did your doctor provide lifestyle guidance (diet, smoking, alcohol, exercise)?** (1) Yes (2) No |  |
| B1.8 | **Did you adjust your lifestyle according to the doctor’s advice?** (1) Yes (2) No |  |
| B1.9 | **Do you receive hypertension follow-up management from primary healthcare institutions?** (1) Yes (2) No (skip to B2) |  |
| B1.10 | **In the past year, how often did primary healthcare institutions follow up for hypertension?** (1) 4 times/year (2) 3 times/year (3) 1–2 times/year (4) None |  |
| B1.11 | **In the past year, what services did they provide during follow-up?** (Multiple choice except option 7) (1) BP measurement (2) Ask about health condition (3) Ask about lifestyle (smoking, alcohol, exercise, diet) (4) Ask about medication use (5) Provide health education/guidance (6) Annual health check (7) None of the above |  |
| **Diabetes** | | |
| B2 | **Have you ever been diagnosed with diabetes by a doctor at a formal medical institution?** (1) Yes (2) No (skip to B3) (9) Don’t know (skip to B3) |  |
| B2.1 | **Year of diabetes diagnosis:** ____ |  |
| B2.2 | **Which measures do you take to control blood sugar?** (Multiple choice except option 1) (1) None (2) Oral hypoglycemic drugs (3) Insulin (4) Dietary control (5) Exercise (6) Blood glucose monitoring (7) Other |  |
| B2.3 | **Have you received guidance from a doctor/nurse on medication use (oral or insulin)?** (1) Yes (2) No |  |
| B2.4 | **If you use medication/insulin, how is your adherence?** (1) Regular (skip to B2.6) (2) Intermittent (3) Do not take/use |  |
| B2.5 | **If not regular, what is the main reason?** (1) Didn’t know necessary (2) Unwilling, think it doesn’t matter (3) Too expensive   1. Side effects/adverse reactions (5) Inconvenient to buy medication   (6) Stop when glucose normalizes (7) Other |  |
| B2.6 | **Do you monitor blood glucose at home?** (1) Never (2) ≥1 time/day (3) 1–6 times/week (4) 1–3 times/month (5) 1–3 times/quarter  (6) 1–3 times/year |  |
| B2.7 | **After diagnosis, did your doctor provide dietary guidance or a meal plan?** (1) Yes (2) No |  |
| B2.8 | **Did you adjust your diet according to the doctor’s advice?** (1) Yes (2) No |  |
| B2.9 | **Do you receive diabetes follow-up management from primary healthcare institutions?** (1) Yes (2) No (skip to B3) |  |
| B2.10 | **In the past year, how often did they follow up for diabetes?** (1) 4 times/year (2) 3 times/year (3) 1–2 times/year (4) None |  |
| B2.11 | **What services did they provide?** (Multiple choice except option 8) (1) BP measurement (2) Ask about health status (3) Ask about lifestyle (4) Ask about medication use (5) Health education/guidance (6) Blood glucose measurement (7) Annual check (8) None |  |
| B2.12 | **In the past year, have you been screened for diabetes complications?** (Multiple choice except option 1) (1) None (2) Foot ulcer (3) Fundus examination (4) Peripheral artery exam (5) Nephropathy screening (6) Neuropathy screening (7) Not sure |  |
| **Hyperlipidemia** | | |
| B3 | **Have you ever been diagnosed with hyperlipidemia by a doctor at a formal medical institution?** (1) Yes (2) No (skip to B4) (9) Don’t know (skip to B4) |  |
| B3.1 | **Year of diagnosis:** ____ |  |
| B3.2 | **Measures taken to control blood lipids:** (Multiple choice except option 1) (1) None (2) Lipid-lowering medication (3) Dietary control (4) Exercise (5) Monitoring (6) Other |  |
| B3.3 | **If you take medication, how is your adherence?** (1) Regular (skip to B4) (2) Intermittent (3) Do not take |  |
| B3.4 | **If not regular, main reason?** (1) Didn’t know necessary (2) Unwilling (3) Too expensive (4) Side effects/adverse reactions (5) Inconvenient to buy medication (6) Stop when lipids normalize (7) Other |  |
| **Other Diseases** | | |
| B4 | **Have you ever been diagnosed with other chronic diseases (besides hypertension, diabetes, hyperlipidemia)?** (1) Yes (2) No (skip to B6) (9) Don’t know (skip to B6) |  |
| B5 | **Have you ever been diagnosed with the following chronic diseases? Please answer each item.**  **a. COPD/bronchitis** (1) Yes (2) No **b. Coronary heart disease** (1 ) Yes (2) No **c. Stroke** (1) Yes (2) No **d. Breast cancer** (1) Yes (2) No **e. Arthritis/rheumatism** (1) Yes (2) No **f. Kidney disease (non-tumor)** (1) Yes (2) No **g. Diabetic retinopathy** (1) Yes (2) No **h. Diabetic foot** (1) Yes (2) No **i. Diabetic neuropathy** (1) Yes (2) No |  |
| B6 | **Do you take aspirin?** (1) Regularly (2) Intermittently (3) Not at all |  |
| B7.1 | **In the past year, have you received influenza vaccination?** (1) Yes (2) No |  |
| B7.2 | **Have you received pneumonia vaccination?** (1) Yes (2) No |  |
| B8.1 | **Have you undergone PCI (stent implantation)?** (1) Yes (2) No (skip to C1) (9) Don’t know (skip to C1) |  |
| B8.2 | **If yes, how many times?** (1) Once (2) Twice (3) Three or more |  |
| **Part III. Behavior and Lifestyle** | | |
| C1 | **Do you smoke?** (Smoking = lifetime ≥100 cigarettes) (1) Yes (2) No (skip to C2) (3) Quit, ___ years |  |
| C1.1 | **Age you started smoking: ____ years** |  |
| C1.2 | **Average cigarettes/day in past month: ____** |  |
| C1.3 | **Did healthcare staff advise you to quit?** (1) No (2) General advice only (3) Advice + treatment plan |  |
| C1.4 | **Compared with previous years, has your smoking changed?** (1) No change (2) Increased (3) Decreased |  |
| C2 | **Do you drink alcohol?** (≥1 time/week in past year) (1) Yes (2) No (skip to C3) (3) Quit, ___ years |  |
| C2.1 | **Age you started drinking: ____ years** |  |
| C2.2 | **Drinking frequency:** (1) Rare (<1 day/week) (2) Occasional (1–2 days/week) (3) Frequent (3–5 days/week)   1. Almost daily (6–7 days/week) |  |
| C2.3 | **Type of alcohol most often consumed (single choice):** (1) Spirits (2) Yellow wine (3) Red wine (4) Beer (5) Rice wine (6) Other |  |
| C2.4 | **Did healthcare staff advise you to quit drinking?** (1) No (2) General advice only (3) Advice + treatment plan |  |
| C2.5 | **Compared with previous years, has your drinking changed?** (1) No change (2) Increased (3) Decreased |  |
| C3 | **Do you perform moderate or above-intensity activities (excluding walking) ≥10 minutes (e.g., cycling, stairs, cleaning)?** (1) Yes (___ times/week, ___ minutes/time) (2) No (skip to C4) |  |
| C4 | **Do you exercise outside of work/farm/household activities (≥10 minutes, moderate/vigorous)?** (1) Yes (2) No (skip to C5) |  |
| C4.1 | **What exercise(s) and duration? (multiple choice)** (1) Walking (___ times/week, ___ min/time) (2) Jogging/brisk walking (___ times/week, ___ min/time) (3) Square dancing (___ times/week, ___ min/time) (4) Tai Chi, etc. (___ times/week, ___ min/time) (5) Other (___ times/week, ___ min/time) |  |
| C5 | **On average, how many hours/day do you spend sitting, reclining, or lying down (excluding sleep)?** ____ hours |  |
| C6 | **On average, how many hours/night do you sleep?** ____ hours |  |
| C6.1 | **In the past month, have you had the following sleep problems? (multiple choice)** (1) Snoring/apnea (2) Difficulty falling asleep (>30 min) (3) Waking ≥2 times/night (4) Use of sleeping pills at least 1 day (5) Early awakening with difficulty returning to sleep  (6) None |  |
| C7 | **Do you usually eat three meals on time?** (1) Yes (2) No |  |
| C7.1 | **Which type of cooking oil do you mainly use?** (1) Animal oil (2) Vegetable oil |  |
