# Supplementary material for: Medication adherence in hypertension and diabetes comorbidity: implications for disease control in a population-based study
Source: Front Public Health. 2026 Jan 30;14:1708587. doi: 10.3389/fpubh.2026.1708587 (PMC12901457; doi:10.3389/fpubh.2026.1708587)
Supplement: Supplementary file 2 [file Table_2.DOCX]

**两慢病患者生活与健康调查问卷**

**□ 单纯高血压患者 □糖尿病患者 □高血压合并糖尿病患者**

您好！欢迎您参与我们的调查！为配合“两慢病”改革工作的开展，我们组织了本次基线调查。请您根据本人的知晓情况如实回答，谢谢！

| 身份证号码：□□□□□□□□□□□□□□□□□□ |
| --- |
| 姓名： 联系电话：_________________ |
| 居住住址： 乡镇（街道） 村（居委会） |
| 调查员签名：___________ 完成日期：_______年____月___日  审核员签名：___________ 完成日期：_______年____月___日 |

| **第一部分 基本信息** | |  |
| --- | --- | --- |
| A1 | 民族： ⑴汉族 ⑵其它 |  |
| A2 | 文化程度： ⑴未接受正规学校教育 ⑵小学及以下 ⑶初中 ⑷高中/中专/技校 ⑸大专 ⑹大学本科及以上 |  |
| A3 | 职业： ⑴离退休 ⑵无业或失业 ⑶家务劳动 ⑷机关、企事业单位管理者 ⑸专业技术人员 ⑹一般办事人员  ⑺商业/服务业人员 ⑻军人 ⑼非农户产业工人 ⑽从事非农劳动的农民 ⑾农业劳动者（从事农林牧渔工作）  ⑿其他从业者 |  |
| A4 | 婚姻状况： ⑴未婚 ⑵在婚 ⑶离婚 ⑷丧偶 |  |
| A5 | 您目前是否独居： ⑴是 ⑵否 |  |
| A6 | 您目前参加了哪种医疗保险？**（选择最主要的一种）**  ⑴城镇职工基本医疗保险 ⑵城乡居民医疗保险 ⑶商业医疗保险 ⑷没参加任何医疗保险 |  |
| A7 | 过去一年，全家人均**年收入**多少元？**（包含家里所有人，包括无收入小孩和共同居住老人）**  ⑴低于10000元 ⑵10000－29999元 ⑶30000－49999元 ⑷50000－79999元 ⑸80000－149999元 ⑹150000元以上 ⑼不回答 |  |
| **第二部分 主要慢性病史及诊疗** | |  |
| B | 您是否有以下疾病家族史？**（除(7)外，其他选项可多选）**  ⑴脑卒中 ⑵糖尿病 ⑶高血压 ⑷高血脂 ⑸心脏病 ⑹其他 （请注明） (7) 无 |  |
| **高血压** | | |
| B1 | 您是否被正规医疗机构医生诊断过高血压？ ⑴是 ⑵否**（跳转到B2）** ⑼不知道**（跳转到B2）** |  |
| B1.1 | 高血压确诊时间： （年） |  |
| B1.2 | 是否采取以下控制血压的措施？**（除⑴外，其他选项可多选）**  ⑴未采取措施 ⑵服用降压药物 ⑶控制饮食 ⑷减少盐的摄入 ⑸增加运动 ⑹血压监测 (7)其他 |  |
| B1.3 | 如服用降压药物，依从性如何？  ⑴规律**（跳转到B1.5）**   ⑵间断 ⑶不服药 |  |
| B1.4 | 如不(规律)服用降压药物，主要原因是什么？  ⑴不知道要服药 ⑵不愿意服药，觉得吃不吃药都差不多 ⑶经济负担重，买不起药 ⑷药物副作用大，服药有不良反应 ⑸买药不方便 ⑹血压正常了就不服药 (7)其他 |  |
| B1.5 | 您在看病时是否有得到过医生或护士在高血压用药方面的指导？ (1)是 (2)否 |  |
| B1.6 | 您平时会自己在家监测血压吗？  ⑴基本不会 ⑵偶尔会测**（少于每天1次）** ⑶每天有规律的测1-3次 ⑷随时会测，每天会测3次以上 |  |
| B1.7 | 您在确诊高血压后是否有医生提供生活方式的指导建议（包括饮食，吸烟，饮酒及体育锻炼等）或具体的指导方案？  (1)是 (2)否 |  |
| B1.8 | 您在患高血压后，是否按照医生的指导对自己的生活习惯（包括饮食，吸烟，饮酒及体育锻炼等）进行了调整？  ⑴是 ⑵否 |  |
| B1.9 | 是否接受基层医疗机构提供的高血压随访管理？ ⑴是 ⑵否**（跳转到B2）** |  |
| B1.10 | 去年一年中，基层医疗机构在给您做高血压相关健康管理时，向您进行随访的频率？  ⑴一年4次 ⑵一年3次 ⑶一年1-2次 ⑷无 |  |
| B1.11 | 去年一年中，基层医疗机构在给您做高血压相关健康管理时，向您提供了下列哪些服务？**（除(7)外，其他可多选）**  ⑴随访测量血压 ⑵随访询问您近期身体情况 ⑶随访询问您的生活方式（如吸烟、饮酒、运动、饮食等） ⑷随访询问您服药情况 ⑸随访对您进行健康教育和健康指导（如生活方式指导） ⑹每年健康体检 (7)没有提供这些服务 |  |
| **糖尿病** | | |
| B2 | 您是否被正规医疗机构医生诊断过糖尿病？ ⑴是 ⑵否**（跳转到B3）** ⑼不知道**（跳转到B3）** |  |
| B2.1 | 糖尿病确诊时间： （年） |  |
| B2.2 | 是否采取以下控制血糖的措施？**（除⑴外，其他选项可多选）**  ⑴未采取措施 ⑵服用降糖药物 ⑶使用胰岛素 ⑷控制饮食  ⑸增加运动 ⑹血糖监测 (7)其他 |  |
| B2.3 | 您在看病时是否有得到过医生或护士在用药方面的指导（包括口服药物和胰岛素注射）？ (1)是 (2)否 |  |
| B2.4 | 如服用降糖药物或使用胰岛素，依从性如何？ ⑴规律**（跳转到B2.6）**   ⑵间断 ⑶不服药或不使用 |  |
| B2.5 | 如不（规律）服用降糖药物或使用胰岛素，主要原因是什么？  ⑴不知道要服药或使用胰岛素 ⑵不愿意服药或使用胰岛素，觉得都差不多 ⑶经济负担重，买不起药或使用胰岛素 ⑷药物或胰岛素副作用大，服药有不良反应 ⑸买药不方便 ⑹血糖正常了就不服药 (7)其他 |  |
| B2.6 | 您平时会自己在家监测血糖吗？  ⑴自己不监测 ⑵每天1次及以上 ⑶每周1-6次 ⑷每月1-3次 ⑸每季度1-3次 ⑹每年1-3次 |  |
| B2.7 | 您在确诊糖尿病后是否有得到医生的饮食指导建议和具体的饮食指导方案？ (1)是 (2)否 |  |
| B2.8 | 您在患糖尿病后，是否按照医生的指导对自己的饮食习惯进行了调整？ ⑴是 ⑵否 |  |
| B2.9 | 是否接受基层医疗机构提供的糖尿病随访管理？  ⑴是 ⑵否**（跳转到B3）** |  |
| B2.10 | 去年一年中，基层医疗机构在给您做糖尿病健康管理时，向您进行随访的频率？  (1)一年4次 (2)一年3次 (3)一年1-2次 (4)无 |  |
| B2.11 | 去年一年中，基层医疗机构在给您做健康管理时，向您提供了下列哪些服务？**（除(8)外，其他选项可多选）**  ⑴随访测量血压 ⑵随访询问您近期身体情况 ⑶随访询问您的生活方式（如吸烟、饮酒、运动、饮食等） ⑷随访询问您服药情况 ⑸随访对您进行健康教育和健康指导（如生活方式指导） ⑹随访测量血糖 (7)每年健康体检 (8)没有提供这些服务 |  |
| B2.12 | 去年一年中，您是否有接受过糖尿病相关并发症的筛查？**（除⑴外，其他选项可多选）**  ⑴无 ⑵糖尿病足溃疡筛查 ⑶糖尿病眼底筛查 ⑷糖尿病下肢动脉血管检查 ⑸糖尿病肾病筛查  ⑹糖尿病相关神经并发症筛查 (7)不清楚 |  |
| **高血脂** | | |
| B3 | 您是否被正规医疗机构医生诊断过血脂异常？ ⑴是 ⑵否**（跳转到B4）** ⑼不知道**（跳转到B4）** |  |
| B3.1 | 血脂异常确诊时间： （年） |  |
| B3.2 | 是否采取以下控制血脂的措施？**（除⑴外，其他选项可多选）**  ⑴未采取措施 ⑵服用调脂药物 ⑶控制饮食 ⑷增加运动 ⑸血脂监测 ⑹其他 |  |
| B3.3 | 如服用调脂药物，依从性如何？ ⑴规律**（跳转到B4）**   ⑵间断 ⑶不服药 |  |
| B3.4 | 如不（规律）服用调脂药物，主要原因是什么？  ⑴不知道要服药 ⑵不愿意服药，觉得吃不吃药都差不多 ⑶经济负担重，买不起药 ⑷药物副作用大，服药有不良反应 ⑸买药不方便 ⑹血脂正常了就不服药 (7)其他 |  |
| **其他疾病** | | |
| B4 | 除高血压、糖尿病和高血脂外，您是否还被正规医疗机构医生诊断过其他慢性病？  ⑴是 ⑵否**（跳转到B6）** ⑼不知道**（跳转到B6）** |  |
| B5 | 您是否被正规医疗机构医生诊断过以下慢性病？ (请在每项中回答，如有，请作进一步说明)   \|  \| **疾病名称** \| **是否患病？** \| **确诊日期？** \| \| --- \| --- \| --- \| --- \| \| a. \| 慢性阻塞性肺部疾病/支气管炎 \| ⑴是 ⑵否**（跳至b）** \|  \| \| b. \| 冠心病 \| ⑴是 ⑵否**（跳至c）** \|  \| \| c. \| 脑卒中 \| ⑴是 ⑵否**（跳至d）** \|  \| \| d. \| 乳腺癌 \| ⑴是 ⑵否**（跳至e）** \|  \| \| e. \| 关节炎或风湿病 \| ⑴是 ⑵否**（跳至f）** \|  \| \| f. \| 肾脏类疾病（不含肿瘤） \| ⑴是 ⑵否**（跳至g）** \|  \| \| g. \| 糖尿病眼病（视网膜病变） \| ⑴是 ⑵否**（跳至h）** \|  \| \| h. \| 糖尿病足 \| ⑴是 ⑵否**（跳至i）** \|  \| \| i. \| 糖尿病相关神经并发症 \| ⑴是 ⑵否**（跳至B6）** \|  \| |  |
| B6 | 您是否服用阿司匹林？ ⑴规律   ⑵间断 ⑶不服药 |  |
| B7.1 | 过去1年，您是否接种过流感疫苗？ ⑴是 ⑵否 |  |
| B7.2 | 您是否接种过肺炎疫苗？ ⑴是 ⑵否 |  |
| B8.1 | 您是否接受过心脏支架手术（PCI）？ ⑴是 ⑵否**（跳转到C1）** ⑼不知道**（跳转到C1）** |  |
| B8.2 | 如果是，请问做过几次心脏支架手术？ ⑴一次 ⑵两次 ⑶三次及以上 |  |
| **第三部分 行为及生活方式** | |  |
| C1 | 您是否吸烟？**（吸烟指的是一生中累计吸烟量超过100支）**  ⑴是 ⑵否**（跳转到C2）** ⑶以前吸，目前已戒烟 年 |  |
| C1.1 | 您开始吸烟的年龄？ （岁） |  |
| C1.2 | 近1个月平均每天吸多少支烟？ （支） |  |
| C1.3 | 在您看病的过程中，是否有医护人员建议您戒烟？  ⑴没有建议 ⑵有建议，仅是一般劝说 ⑶有建议，且提供了治疗方案（行为或药物） |  |
| C1.4 | 您现在的吸烟量和之前相比是否有明显变化？  ⑴变化不大 ⑵较往年明显增加 ⑶较往年明显减少 |  |
| C2 | 您是否饮酒？**（饮酒指的是在过去一年内，每周至少饮酒1次）**  ⑴是 ⑵否**（跳转到C3）** ⑶以前饮酒，目前已戒酒 年 |  |
| C2.1 | 您开始饮酒的年龄？ （岁） |  |
| C2.2 | 您的饮酒频率如何？  ⑴很少(少于1天/周) ⑵偶尔(1-2天/周) ⑶经常(3-5天/周) ⑷几乎每天(6-7天/周) |  |
| C2.3 | 您最常喝什么酒？**（限单选）**  ⑴白酒 ⑵黄酒 ⑶红酒 ⑷啤酒 (5)米酒 ⑸其它 |  |
| C2.4 | 在您看病的过程中，是否有医护人员建议您戒酒？   1. 没有建议 ⑵有建议，仅是一般劝说 ⑶有建议，且提供了戒酒方案 |  |
| C2.5 | 您现在的饮酒量和之前相比是否有明显变化？  ⑴变化不大 ⑵较往年明显增加 ⑶较往年明显减少 |  |
| C3 | 您的工作、农活及家务活动中，有没有中等及以上强度活动（**除步行以外的其他活动，如骑自行车上下班，爬楼梯，擦窗户等**），并且活动时间持续10分钟以上？  ⑴有（每周 次/每次 分钟） ⑵没有**（跳转到C4）** |  |
| C4 | 除工作、农活及家务活动外，您是否进行体育锻炼？**（持续至少10分钟引起呼吸、心跳增加的中高强度活动，如快步走、打太极拳、长跑、游泳、踢足球等）** ⑴是 ⑵否**（跳转到C5）** |  |
| C4.1 | 您最常用哪种锻炼方式及其锻炼时间？**（多选）**  ⑴散步 （每周 次/每次 分钟）  ⑵慢跑或快步走（每周 次/每次 分钟）  ⑶跳广场舞 （每周 次/每次 分钟）  ⑷太极拳等 （每周 次/每次 分钟）  ⑸其他 （每周 次/每次 分钟） |  |
| C5 | 通常一天内，您坐着、靠着或躺着（包括坐着工作、学习、看电视、用电脑、阅读、娱乐、休息等，但不包括睡觉时间）的累计时间是多少？ （小时） |  |
| C6 | 通常一天内，您晚**上**睡觉累计有多少时间？ （小时） |  |
| C6.1 | 近1个月您是否有以下睡眠问题？**（可多选）**  ⑴打鼾或窒息、憋气 ⑵入睡困难（入睡时间30分钟以上） ⑶中间觉醒两次以上（含两次）  ⑷至少有1天服用安眠药（西药或中药）以帮助睡眠 (5)早醒，并难以重新入睡 ⑹无睡眠问题 |  |
| C7 | 通常您一日三餐是按时的吗? ⑴是 ⑵否 |  |
| C7.1 | 您通常食用的油以哪种为主： ⑴动物油 ⑵植物油 |  |
